# Perception, attitude, knowledge and learning style preference on challenges of antimicrobial resistance and antimicrobial overuse among first year doctors in training and final year medical students

**DOI:** 10.1186/s13756-019-0597-0

**Published:** 2019-08-23

**Authors:** Pinyo Rattanaumpawan, Nuttagarn Chuenchom, Visanu Thamlikitkul

**Affiliations:** Division of Infectious Diseases and Tropical Medicine, Department of Medicine, Faculty of Medicine Siriraj Hospital, Mahidol University, 2 Wang Lang Rd., Bangkok Noi, Bangkok, Thailand

**Keywords:** Antimicrobial overuse, Antimicrobial resistance, Attitude, Learning style preference, Medical education, Perception, Questionnaire

## Abstract

**Background:**

To promote effective education on challenges of antimicrobial resistance (AMR) and antimicrobial overuse, it is necessary to understand the current perception, attitude, knowledge and learning style preference on these issues among future general practitioners and subspecialists.

**Methods:**

In 2014, we conducted a questionnaire-based survey in two participant groups: 1) first-year residents and fellows (doctor-in-training, DIT) of Faculty of Medicine Siriraj Hospital, Mahidol University, Thailand and 2) final-year medical students (medical students, MS) of three medical schools in Thailand.

**Results:**

A total of 225 DIT and 455 MS completed the questionnaire. Both groups had good perception of these problems. However, overall, only half of the participants answered that they can prescribe appropriate antibiotics to their patients (DIT 48.4% vs. MS 52.8%; *p* = 0.29). The DIT group had significantly higher mean knowledge scores on questions relating to antimicrobial use (64.0% vs. 56.0%; *p* < 0.001) and infection control (83.0% vs. 80.0%; *p* = 0.004). For the DIT group, the learning style preference regarding antimicrobial use was conducting an internet search (56.0%), followed by asking infectious disease personnel (50.7%) and/or using the Thai-language antimicrobial guidelines. By contrast, the MS group preferred asking ward personnel (57.4%), followed by using the English-language antimicrobial guidelines (52.1%) and conducting an internet search (27.7%).

**Conclusion:**

AMR and antimicrobial overuse in Thailand are well recognized challenges. However, final-year MS and first-year DIT have very limited knowledge of these important subjects. Customized education methods should be carefully chosen to ensure that future general practitioners and subspecialists are adequately trained.

## Background

Antimicrobial resistance (AMR) has been mainly attributed to antimicrobial overuse. The problem of antimicrobial overuse is driven by multiple factors including prescriber’s lack of knowledge and poor attitude and insufficient diagnostic tools and monitoring systems [1–3]. The knowledge of rational antimicrobial use was found to be important, while extrinsic factors such as patients or the healthcare system were found to be less important [4]. A recent systemic review identified six intrinsic attitude components that play an influential role in indiscreet antimicrobial prescription by physicians leading to AMR: complacency, fear of future complications, ignorance, indifference to positive and negative motivation in antimicrobial prescription, responsibility or belief and confidence [5].

Appropriate antimicrobial use has become standard practice in all clinical settings and is strongly emphasized in undergraduate and postgraduate medical training [6]. To ensure effective education on the challenges of AMR and antimicrobial overuse, it is necessary to understand the current perception, knowledge and learning style preference on these issues among prescribers.

A previous 24-item electronic survey on antimicrobial prescription and education in future prescribers (fourth-year medical students) conducted in the United States revealed good attitude, high recognition of the importance of appropriate antimicrobial use and eagerness to learn more on rational antimicrobial use. However, only half of the respondents knew how to appropriately prescribe antimicrobial treatment in patients with upper respiratory tract infection [7]. A recent questionnaire survey conducted in Thailand reported similar results. Among Thai medical students, AMR is a well-recognized challenge, however, their knowledge of AMR and appropriate antimicrobial use was considerably limited [8].

Despite the substantial research on undergraduate medical training of AMR and antimicrobial overuse [3, 7–12], only a few studies on post-graduate medical training have been published [12–14]. Given these considerations, we conducted a questionnaire-based survey among doctors-in-training (first-year residents and fellows) with the aim to determine perception, attitude, knowledge and learning style preference on challenges of AMR and antimicrobial overuse. Results of this survey were subsequently compared with the results from the previous survey conducted among' final-year Thai medical students.

## Methods

### Study design and setting

This study comprised two parts: 1) doctor-in-training (DIT) survey, a questionnaire-based survey among first-year residents (or postgraduate training year-1) and first-year fellows (doctors who already completed their residency training and currently in the subspecialty training) at the Faculty of Medicine Siriraj Hospital, Mahidol University, Bangkok, Thailand; and 2) medical student (MS) survey, a questionnaire-based survey among final-year medical students at three medical schools in Thailand (Faculty of Medicine Siriraj Hospital, Mahidol University; Faculty of Medicine, Chiangmai University; and Faculty of Medicine, Naresuan University). These three medical schools were selected because of their convenient accessibility. Complete results of the MS survey have been published elsewhere [8].

Data from both DIT and MS surveys were subsequently compared and analyzed.

The study protocol was approved by the Institutional Review Boards of all institutions, which waived the need for informed consent from participants.

### Questionnaire development

The study questionnaire was specifically developed to obtain the necessary information regarding perception, attitude, knowledge and learning style preference on AMR and antimicrobial overuse, as well as participants’ baseline characteristics. The questionnaire included closed- and open-ended questions and questions using a 5-point Likert scale (5 = strongly agree, 4 = agree, 3 = neutral, 2 = disagree and 1 = strongly disagree). Good perception or attitude was noted if a participant agreed or strongly agreed (scale 4–5) in a positive question or disagreed or strongly disagreed (scale 1–2) in a negative question.

The perception and attitude assessment section included 12 questions relating to AMR, appropriate antimicrobial use and infection control and 10 questions relating to knowledge and preparedness of antimicrobial use.

The knowledge assessment section included 5 questions about mechanisms of AMR (e.g. what is the main AMR mechanism of methicillin resistant *Staphylococcus aureus*?, seven clinical scenarios with common infection problems (e.g., common cold and acute diarrhea) and 5 questions about appropriate infection control procedures for several types of situation (e.g. how to manage a patient with MRSA or pulmonary TB.)

The assessment of learning style preference asked about preferred self-study methods to access scientific information.

The baseline characteristic section included questions regarding sex, age and grade point average (GPA) during attainment of their medical degree. The DIT questionnaire also included questions relating to the participant’s training department and previous workplaces.

### Questionnaire distribution

The DIT questionnaire was distributed to all first-year residents and fellows during their first orientation, which was conducted in June 2014. The MS questionnaire was distributed to all final-year medical students at their institution’s last orientation (March–April 2014). Participation was voluntary and anonymous. After completing the questionnaire, the participants were given an entry into a raffle, in which there were 10 prizes worth a total value of 35,000 Thai baht (or approximately 850 British pounds).

### Statistical analysis

We assumed the prevalence of choice selections in each question varied from 10 to 50%.

By using an allowable error of 20% and a two-sided alpha error of 0.05, a sample size of 150 per group was required. In 2014, there were approximately 300 first-year residents and fellows in the post-graduate training program at the Faculty of Medicine Siriraj Hospital and 600 final-year medical students enrolled at the three medical schools. We assumed a response rate of > 50%, thus we would be able to reach an adequate sample size.

Categorical variables are reported as frequencies and percentages. Continuous variables are reported as mean ± standard deviations (SD) and medians and ranges according to the distribution. Student’s t-test and chi-square test were used to assess differences between groups (DIT vs. MS). All analyses were performed using STATA version 14.0 (STATA Corp, College Station, TX, USA). A two-sided *p*-value of < 0.05 was considered statistically significant.

## Results

A total of 680 participants completed the questionnaire: 225 participants in the DIT group and 455 participants in the MS group. The response rate of the DIT group and the MS group was 78.9% (225/285) and 71.5% (455/636), respectively. Missing data were noted in 0.3% of all questions.

### Baseline characteristics

More than half of the participants in both groups (DIT vs. MS; *p*-value) were female (63.1% vs. 57.1%; *p* = 0.14). The DIT group had a significantly higher mean age ± SD (26.71 ± 1.22 vs. 24.51 ± 2.24; *p* < 0.001) and mean GPA (3.31 ± 0.29 vs. 3.20 ± 0.35; p < 0.001). The three leading training subspecialties of the DIT group were Internal Medicine (26.7%), Radiology (15.6%) and Pediatrics (10.7%). More than half of the DIT group previously worked in a tertiary care hospital (53.8%) and/or private hospital (63.6%) before entering the training program. Details of participants’ baseline characteristics are shown in Table 1.

**Table 1:** Baseline characteristics of participants in DIT and MS groups

Baseline characteristics	DIT (*N* = 225)	MS (*N* = 455)	*p*-value
Sex, female (%)	142 (63.1%)	260 (57.1%)	0.14
Mean age ± SD	26.71 ± 1.22	24.51 ± 2.24	< 0.001
Mean GPA ± SD	3.31 ± 0.29 (*n* = 214)	3.20 ± 0.35 (*n* = 401)	< 0.001
Training specialties			
Internal Medicine	60 (26.7%)	NA	NA
Radiology	35 (15.6%)	NA	NA
Pediatrics	24 (10.7%)	NA	NA
Anesthesiology	20 (8.9%)	NA	NA
Surgery	19 (8.4%)	NA	NA
Others^a^	67 (29.8%)	NA	NA
Previous workplaces (may select more than one answer)			
University hospital	25 (11.1%)	NA	NA
Tertiary care hospital	121 (53.8%)	NA	NA
Secondary care hospital	2 (0.9%)	NA	NA
Primary care hospital or center	58 (25.8%)	NA	NA
Private hospital	143 (63.6%)	NA	NA
Other type of medical facility^b^	10 (4.4%)	NA	NA

*NA* Not applicable

^a^Includes orthopedic surgery, ophthalmology, otolaryngology and rehabilitation

^b^Includes private clinics, health insurance companies, etc.

### Perception and attitude on AMR and antimicrobial overuse

The majority of both groups (DIT vs. MS; *p*-value) had good perception that inappropriate use of antimicrobials can harm patients (94.2% vs. 95.4%; *p* = 0.51) and prescribing broad-spectrum antimicrobial agents increases AMR (94.7% vs. 89.7%; *p* = 0.03). However, only half of both groups well perceived that appropriate use of antimicrobials can also cause AMR (47.1% vs. 48.8%; *p* = 0.68). The DIT group had a significantly higher proportion of participants with good perception that antimicrobial overuse (94.7% vs. 85.1%; *p* < 0.001) and AMR (92.4% vs. 84.6%; *p* = 0.004) are considered national issues. Details on perception and attitude on AMR and antimicrobial overuse are presented in Table 2.

**Table 2:** Perception and attitude on appropriate antimicrobial use, antimicrobial resistance and infection control between DIT and MS groups

Perceptions and attitudes	Mean Likert scale score (±SD)		*p*-value	Good perception^a^ (%)		*p*-value
	DIT (*N* = 225)	MS (*N* = 455)		DIT (*N* = 225)	MS (*N* = 455)	
1. Inappropriate use of antimicrobials can harm patients	4.58 ± 0.63	4.52 ± 0.59	0.29	94.2%	95.4%	0.51
2. Prescribing broad-spectrum antimicrobials increases antimicrobial resistance	4.54 ± 0.65	4.45 ± 0.71	0.12	94.7%	89.7%	0.03
3. Appropriate use of antimicrobials can cause antimicrobial resistance	3.37 ± 0.88	3.43 ± 0.87	0.35	47.1%	48.8%	0.68
4. Antimicrobials are overused in our hospitals	3.84 ± 0.88	3.94 ± 0.87	0.19	65.3%	71.0%	0.13
5. Antimicrobials are overused nationally	4.46 ± 0.63	4.26 ± 0.75	< 0.001	94.7%	85.1%	< 0.001
6. Antimicrobial resistance is not a significant problem in our hospital^b^	2.16 ± 0.93	2.10 ± 0.98	0.51	73.3%	75.4%	0.56
7. Antimicrobial resistance is not a significant problem nationally^b^	1.73 ± 0.70	1.91 ± 0.88	0.008	92.4%	84.6%	0.004
8. New antimicrobials will be developed in the future to solve antimicrobial resistance	4.26 ± 0.74	4.37 ± 0.71	0.05	86.2%	89.0%	0.29
9. Poor adherence to hand hygiene practices can cause the spread of antimicrobial resistance among patients	4.41 ± 0.72	4.40 ± 0.67	0.91	89.8%	91.2%	0.55
10. I would like more education on how to use antimicrobials appropriately	4.43 ± 0.65	4.51 ± 0.63	0.08	92.0%	93.6%	0.43
11. I would like more education on antimicrobial resistance	4.35 ± 0.69	4.46 ± 0.66	0.04	89.3%	91.4%	0.38
12. I would like more education on hospital infection control	4.28 ± 0.69	4.32 ± 0.69	0.44	88.4%	89.5%	0.69

^a^Good perception was noted if a participant agreed or strongly agreed (scale 4–5) in a positive question or disagreed or strongly disagreed (scale 1–2) in a negative question ^b^Questions 6 and 7 are negative questions. All other questions are positive questions

### Perception and attitude on knowledge and preparedness of antimicrobial use

Only one-third of both groups (DIT vs. MS; *p*-value) well perceived that they received adequate training on appropriate antimicrobial use (32.0% vs. 39.6%; *p* = 0.06) and approximately half of both groups well perceived that they can prescribe appropriate antibiotics to their patients (48.4% vs. 52.8%; *p* = 0.29). A significantly higher proportion of participants in the DIT group agreed or strongly agreed that antimicrobials should not be prescribed for treatment of common cold (75.1% vs. 59.1%; *p* < 0.001) and food poisoning (74.7% vs. 56.7%; p < 0.001) and did not feel anxious when prescribing antimicrobial agents (53.3% vs. 28.6%; p < 0.001). Surprisingly, a significantly lower proportion of participants in the DIT group perceived that they can access reliable sources of information on antimicrobial use (53.8% vs. 65.5%; *p* = 0.003), know how to prevent and control spread of AMR (48.4% vs. 64.8%; *p* < 0.001) and know when they have to wear a surgical mask for routine patient care (59.6% vs. 77.4%; *p* < 0.001). Details of perception and attitude on knowledge and preparedness of antimicrobial use are presented in Table 3.

**Table 3:** Perception and attitude on knowledge and preparedness of antimicrobial use between DIT and MS groups

Perceptions and attitudes	Mean Likert scale score (±SD)		*p*-value	Good perception^a^ (%)		*p*-value
	DIT (*N* = 225)	MS (*N* = 455)		DIT (*N* = 225)	MS (*N* = 455)	
1. I have been adequately trained in the appropriate use of antimicrobials	3.12 ± 0.77	3.30 ± 0.77	0.005	32.0%	39.6%	0.06
2. I can access reliable sources for knowledge on antimicrobial use	3.48 ± 0.67	3.76 ± 0.72	< 0.001	53.8%	65.5%	0.003
3. I know which patient needs to be treated with antimicrobials	3.82 ± 0.55	3.90 ± 0.63	0.11	74.7%	75.4%	0.84
4. I can prescribe the appropriate antimicrobials to patients	3.47 ± 0.60	3.57 ± 0.64	0.06	48.4%	52.8%	0.29
5. We should prescribe antimicrobial agents to patients with symptoms of fever, cough, sore throat and runny nose^b^	2.03 ± 0.76	2.42 ± 0.93	< 0.001	75.1%	59.1%	< 0.001
6. We should prescribe antimicrobial agents to patients with diarrhea and vomiting from food poisoning^b^	2.09 ± 0.84	2.44 ± 0.96	< 0.001	74.7%	56.7%	< 0.001
7. I feel anxious when I have to prescribe antimicrobial agents^b^	2.52 ± 0.74	2.99 ± 0.84	< 0.001	53.3%	28.6%	< 0.001
8. I am aware of appropriate antimicrobial use in routine patient care	4.17 ± 0.67	4.19 ± 0.68	0.63	85.3%	86.2%	0.77
9. I know how to prevent and control spread of antimicrobial resistance .	3.50 ± 0.65	3.75 ± 0.72	< 0.001	48.4%	64.8%	< 0.001
10. I know when I have to wear a surgical mask for routine patient care	3.61 ± 0.60	4.02 ± 0.73	< 0.001	59.6%	77.4%	< 0.001

^a^Good perception was noted if a participant agreed or strongly agreed (scale 4–5) in a positive question or disagreed or strongly disagreed (scale 1–2) in a negative question

^b^Questions 5–7 are negative questions. All other questions are positive questions

### Knowledge on mechanism of AMR, antimicrobial use and infection control

**Fig. 1:**
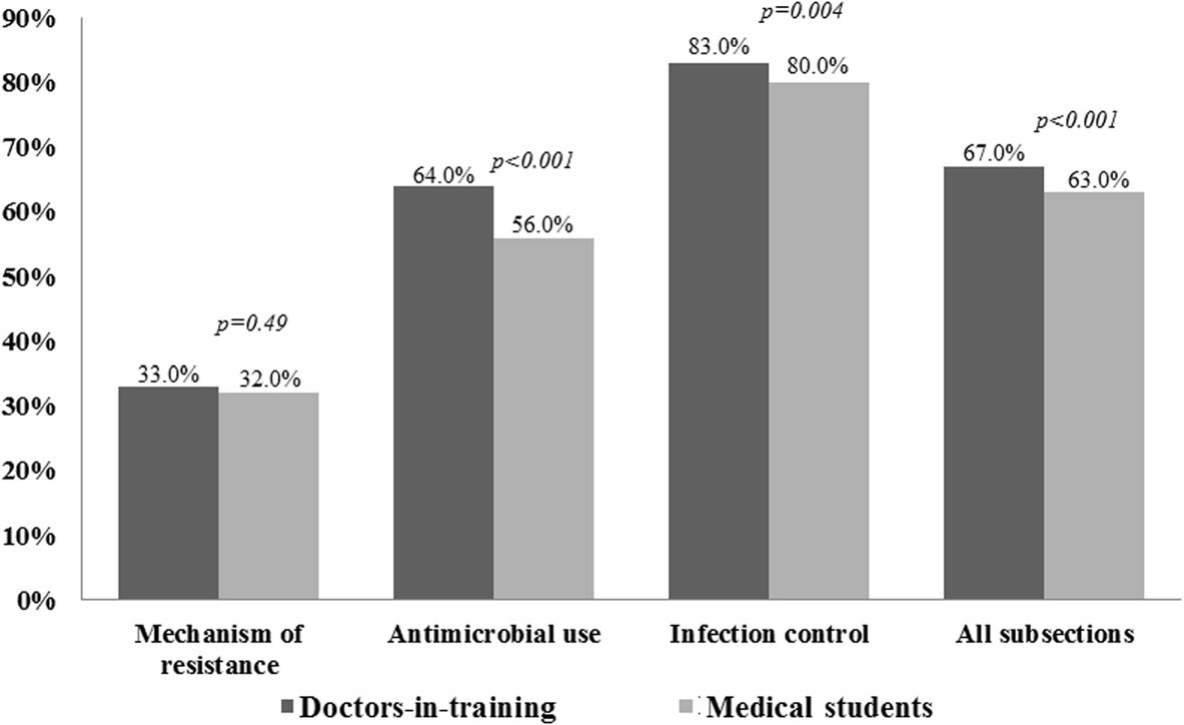
Mean knowledge scores of doctors-in-training and medical students. Significant differences between groups were determined using the chi-square test

Mean knowledge scores on questions relating to mechanism of AMR among DIT and MS groups were comparable (33.0% vs. 32.0%; *p* = 0.49). However, the DIT group had significantly higher mean knowledge scores on questions relating to antimicrobial use (64.0% vs. 56.0%; *p* < 0.001), infection control (83.0% vs. 80%; *p* = 0.004) and overall (67.0% vs. 63.0%; p < 0.001). Knowledge scores for each subsection are shown in Fig. 1.

### Learning style preference

**Fig. 2:**
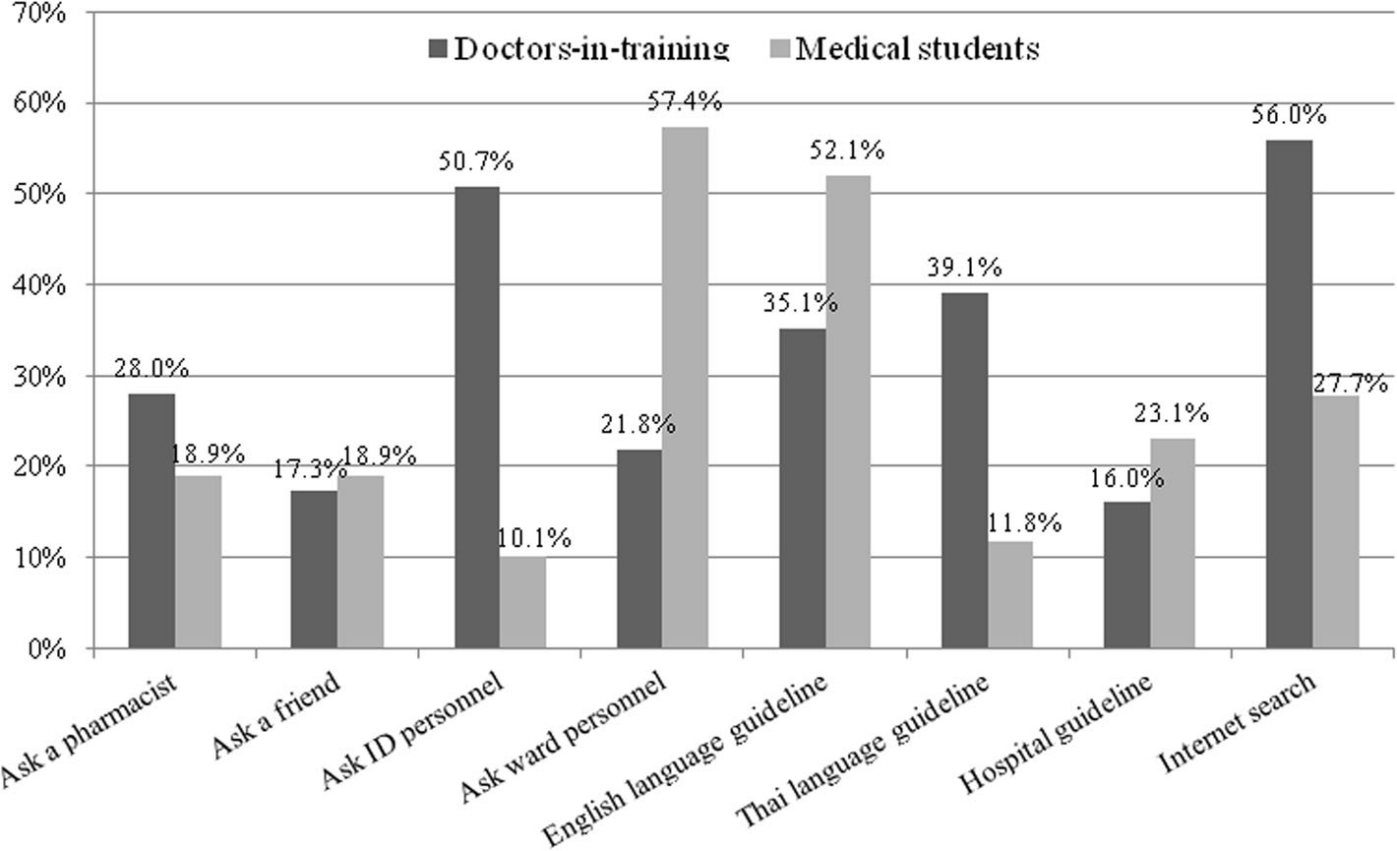
Learning style preferences regarding antimicrobial use of doctors-in-training and medical students. Significant differences were observed between the two groups for all learning styles (chi-square test, *p* < 0.001 for all)

If participants in the DIT group had any questions regarding antimicrobial use, they preferred conducting an internet search (56.0%), followed by asking infectious disease (ID) personnel (50.7%) and/or using the Thai-language antimicrobial guidelines. By contrast, participants in the MS group preferred asking ward personnel (57.4%), followed by using the English-language antimicrobial guidelines (52.1%) and conducting an internet search (27.7%). Approximately 10% of participants in the MS group preferred to ask ID personnel or use the Thai language guidelines. The preference of all learning styles was significantly different between the two groups (*p* < 0.001). Figure 2 shows participants’ learning style preference regarding antimicrobial use for both DIT and MS groups.

## Discussion

Participants in both groups had good perception of the challenges of AMR and antimicrobial overuse. Similar to previous studies [3, 7–12], the majority of participants also recognized the problem of antimicrobial overuse at the national level, but only 75% of them recognized that AMR is also an issue at their hospitals. Furthermore, a significant proportion of participants felt that they still needed further education on AMR, rational antimicrobial use and infection control. This is considered a great obstacle to reducing inappropriate antimicrobial use.

Participants in the DIT group were more likely to have good perception for nearly all questions regarding knowledge of antimicrobial use compared with those in the MS group. However, the DIT group was less likely to report that they knew when to control the spread of AMR and when to wear a surgical mask for routine patient care. Due to growing concerns regarding the spread of multidrug resistant organisms over the recent years, it is reasonably to believe that the MS group may receive better undergraduate education on infection control compared with the DIT group. These findings emphasized the urgent need for post-graduate medical training to focus on infection prevention control measures.

Participants in both groups achieved low scores in the knowledge section, especially in the mechanism of AMR subsection. As expected, the DIT group had a significantly higher score in nearly all subsections than the MS group. In detail, approximately half of participants in both groups misunderstood that antimicrobial agents are necessary for patients with asymptomatic bacteriuria as well as routine episiotomy.

Notably, the learning style preference regarding antimicrobial use in DIT and MS groups completely differed. While the DIT group preferred to ask ID personnel, the MS group preferred to ask ward personnel. This may be explained by the complex relationship between the supervisor and trainee. Ward personnel, who spend more time in the ward, may be regarded to be more approachable by MS than ID personnel. Conversely, DIT may perceive that ID personnel are more reliable in providing rational antimicrobial recommendations than ward personnel, who are not ID specialists. Unfortunately, our study did not thoroughly explore the rationale for each learning style preference. By the way, good communication between MS and ID personnel should be encouraged.

Although approximately 40% of the DIT group preferred to use the Thai-language antimicrobial guideline, only 10% of the MS group preferred to use the Thai-language antimicrobial guidelines. This may result from the lack of a printed version of the Thai-language antimicrobial guidelines. Most Thai-language antimicrobial guidelines are available online on the professional medical association website, which is not widely known.

Our study has some potential limitations. First, participation bias may be an issue. Participants with good perception, attitude and knowledge may be more likely to participate. To minimize this issue, we distributed the DIT questionnaire during the first orientation, during which > 70% of participants were likely to attend. Furthermore, we distributed the MS questionnaire at the last orientation, during which > 90% of participants were likely to attend. Second, the study was conducted at university hospitals; therefore, generalizability may be an issue. Results from this study may not be applicable to students studying at community hospitals. Lastly, we collected data via a questionnaire, which inevitably results in some missing data, especially personal information.

Our study also has some strengths that must be mentioned. First, our study did not only focus on perception, attitude and knowledge, but also evaluated learning style preference on AMR and antimicrobial overuse. Second, our study enrolled two important target groups: future general practitioners (final-year medical students) and future subspecialists (first-year residents and fellows). Comparison between these two groups allows us to clearly understand the complexity of learning style preferences across generations.

## Conclusion

Challenges of AMR and antimicrobial overuse in Thailand are well-recognized by both future general practitioners and subspecialists. However, their knowledge of these important subjects is substantially limited. Customized education methods should be carefully chosen to ensure that they have access to all vital knowledge regarding AMR and skills in rational antimicrobial use and appropriate infection control before completion of their training.

## Data Availability

Data is available upon request.
